# Visual inspection provides precise intraoperative evaluation of tibial slope during unicompartmental knee arthroplasty

**DOI:** 10.1002/ksa.70169

**Published:** 2025-11-03

**Authors:** Harun Hawi, Humam Hawi, Emmanouil Liodakis, Nael Hawi, Rüdiger von Eisenhart‐Rothe, Georg Matziolis

**Affiliations:** ^1^ Orthopaedics University Hospital Jena, Campus Eisenberg, Friedrich‐Schiller‐University, Waldkliniken Eisenberg Eisenberg Thuriniga Germany; ^2^ Department of Trauma, Hand and Reconstructive Surgery Saarland University Hospital Homburg Saarland Germany; ^3^ Orthopaedic and Surgical Clinic Braunschweig (OCP) Braunschweig Lower Saxony Germany; ^4^ Department of Orthopedics TUM‐Universitätsklinikum Rechts der Isar, Technical University Munich München Bavaria Germany

**Keywords:** accuracy, intraoperative assessment, posterior tibial slope (PTS), unicompartmental knee arthroplasty (UKA), visual inspection

## Abstract

**Purpose:**

Accurate reconstruction of the posterior tibial slope (PTS) is essential for successful unicompartmental knee arthroplasty (UKA), yet intraoperative assessment remains challenging without advanced navigation tools. This study evaluated the accuracy of visual intraoperative assessment of PTS changes after tibial resection.

**Methods:**

Resection blocks from total knee arthroplasty (TKA) were processed to simulate UKA resections, providing a wide range of slope variations. PTS changes were measured radiographically and via optical scans of resection blocks from 55 patients with severe osteoarthritis. Three experienced surgeons visually estimated PTS changes, which were compared to reference measurements using intraclass correlation coefficients (ICCs), Pearson correlation, analysis of variance (ANOVA) and Tukey's honestly significant difference (HSD) tests.

**Results:**

Optical scan and radiographic measurements showed near‐perfect agreement (ICC = 0.99). The mean deviation between surgeon estimates and radiographic measurements was 1.0° ± 0.7° (range 0°–3.0°). Examiner ICCs ranged from 0.88 to 0.96, and Pearson correlations were strong (0.77–0.87). ANOVA and HSD tests showed no significant differences between visual and reference measurements.

**Conclusion:**

Visual inspection by experienced surgeons provides a sufficiently accurate and reliable method for intraoperative assessment of PTS during UKA.

**Level of Evidence:**

Level IV.

**Abbreviations**
ANOVA: analysis of variance
HSD: honestly significant difference
ICC: intraclass correlation coefficient
PTS: posterior tibial slope
ROM: range of motion
TKA: total knee arthroplasty
UKA: unicompartmental knee arthroplasty

## INTRODUCTION

Unicompartmental knee arthroplasty (UKA) has become an increasingly popular option for patients with isolated medial or lateral compartment osteoarthritis, offering a less invasive alternative to total knee arthroplasty (TKA) [3]. Advantages of UKA include preservation of the cruciate ligaments, maintenance of more physiological knee kinematics, faster recovery and reduced perioperative morbidity compared with TKA [15, 20].

A critical technical factor in UKA is the correct reconstruction of the posterior tibial slope (PTS). The PTS influences sagittal stability, tibial translation and cruciate ligament tension, and importantly affects achievable flexion and extension gaps [11, 21]. Altering the PTS—whether by steepening or flattening relative to the native slope—may lead to restricted extension or flexion, respectively, thereby compromising range of motion (ROM) and clinical outcomes [12, 23, 26]. Indeed, deviations from the native slope have been implicated in implant instability, suboptimal kinematics and component failure [11, 13, 14].

Despite its importance, no standardised method exists for assessing PTS intraoperatively in manual UKA without navigation or robotics. While radiographic or navigation‐based approaches improve accuracy, they are time‐consuming, expensive and not routinely accessible [7]. Alternative referencing techniques, such as the use of a tibial plateau probe, have shown improved reproducibility [1, 2]. Nevertheless, some surgeons rely on visual inspection of the tibial resection surface, though its accuracy and reliability remain unvalidated.

This study aimed to quantify the accuracy of visual PTS assessment by experienced surgeons compared with radiographic and optical measurements. We hypothesised that visual estimation produces minimal error and can reliably identify clinically relevant deviations, offering a practical intraoperative solution when advanced systems are unavailable.

## METHODS

This retrospective study analysed 55 consecutive patients who underwent TKA for advanced osteoarthritis (Kellgren–Lawrence Grade ≥III) in 2020. All patients had previously received unsuccessful conservative treatment. Resection blocks with under‐resections were excluded, and no restrictions were applied regarding age or sex. All procedures were performed by board‐certified orthopaedic surgeons at the Waldkliniken Eisenberg Hospital using extramedullary alignment instruments and preoperative radiographic planning. Ethical approval was obtained from the local committee (Reg.‐Nr.: 2020‐2019_2‐Material).

TKA resections were selected instead of UKA resections to achieve a wider range of PTS variations, thereby allowing a more robust evaluation of visual estimation accuracy.

**Figure 1:**
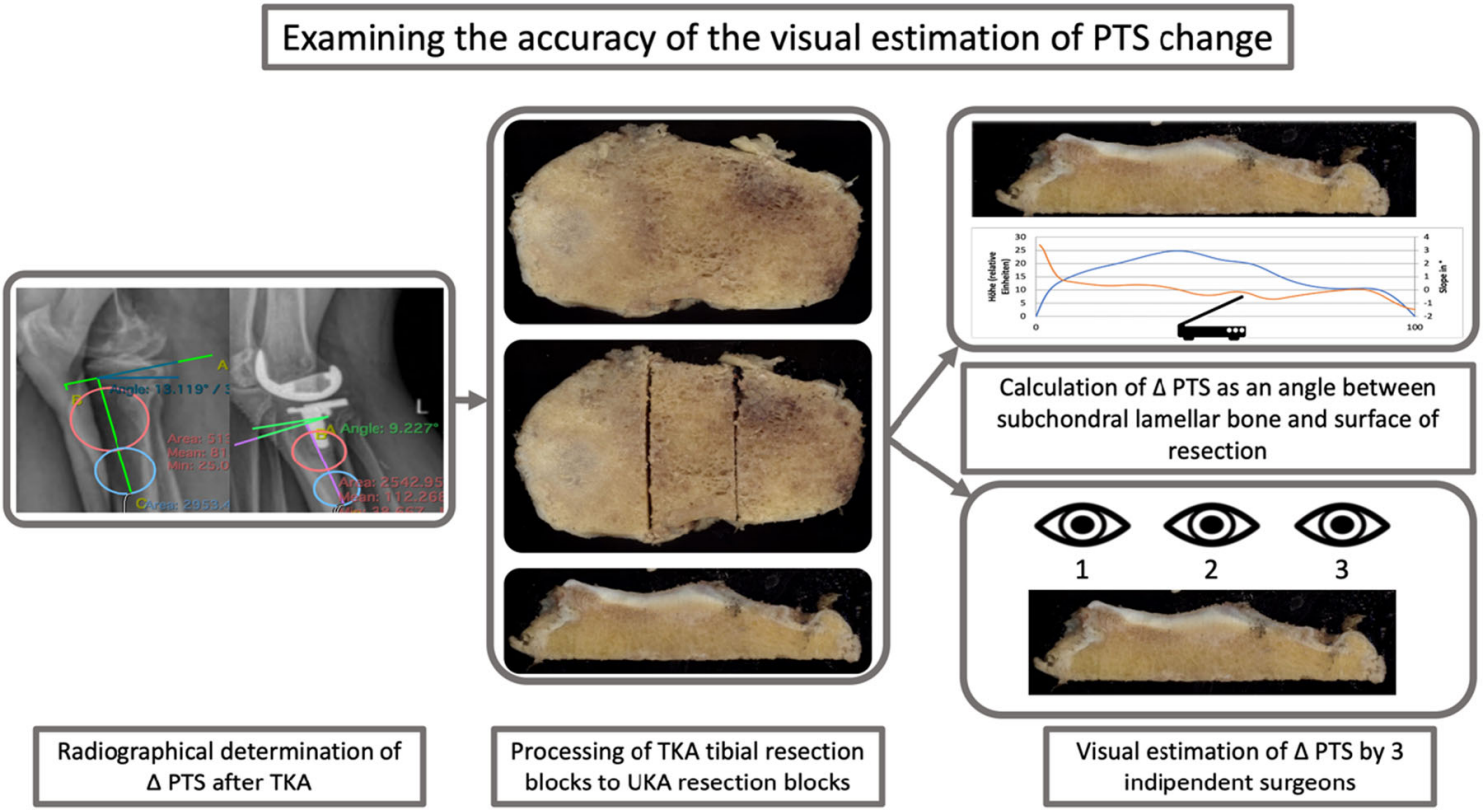
Study protocol. PTS, posterior tibial slope; TKA, total knee arthroplasty; UKA, unicompartmental knee arthroplasty.

Immediately after surgery, the tibial resection blocks were fixed in 10% formalin and stored at 27°C for approximately 1 week. To simulate UKA resections, the blocks were trimmed with a band saw, as illustrated in Figure 1. Each block was scanned using a flatbed scanner, and the anterior–posterior orientation was marked. Fifteen evenly distributed points along the cortical bone were identified using ImageJ software to minimise the influence of cartilage loss. The tibial slope was then calculated by fitting spline functions to these points and determining the mean slope in the central joint area. This automated method eliminated examiner bias and provided an independent reference for validation.

Radiographic measurements were obtained pre‐ and postoperatively with the EOS Imaging System in full weight‐bearing position. PTS was measured following the technique described by Jahn et al. [10], defining the tibial axis through two concentric circles along the shaft and calculating the slope relative to this axis.

Each resection block was anonymised and randomly ordered. Three experienced orthopaedic surgeons (>150 UKA procedures each) independently estimated the change in PTS to the nearest degree, blinded to the scan and radiographic results.

### Sample size calculation

The minimum required sample size was determined using an expected intraclass correlation coefficient (ICC) of 0.90, a null hypothesis ICC of 0.70, *α* = 0.05 and power = 0.80. This yielded a requirement of 36 samples; the inclusion of 55 resections ensured adequate statistical power.

### Statistical analysis

All data were analysed using the statistical program “SPSS” (SPSS, IBM Version 12.0). The ICC and Pearson's correlation coefficient were selected for statistical analysis due to their ability to assess the reliability and strength of the relationship between different measurement methods.

The ICC was chosen to evaluate the consistency and agreement between different measurements, specifically between the visual assessments of the surgeons and the radiographic and optical scan measurements. The ICC is especially useful when evaluating the consistency of ratings provided by multiple raters or measurement techniques. A high ICC value indicates that the visual assessments align not only in terms of correlation but also in their consistency, ensuring that the visual method can be reliably used for estimating changes in PTS during surgery.

The ICC was calculated for both intra‐ and interobserver reliability. An ICC of <0.5 was considered poor agreement, a value between 0.5 and 0.75 was considered moderate agreement, and values above 0.76 were considered high agreement. If the ICC exceeded 0.90, excellent agreement was assumed. Correlations between the results of the different measurement techniques were tested by calculating the Pearson correlation coefficient.

Pearson's correlation coefficient was selected to assess the strength and direction of the linear relationship between the visual estimates of the surgeons and the reference measurements. Pearson's coefficient is appropriate when evaluating the degree of correlation between two continuous variables, quantifying how closely the changes in PTS measured by one method (e.g., visual estimates) are associated with those measured by another method (e.g., radiographs or scans). A strong positive correlation would indicate that the surgeons' visual estimates are closely aligned with the reference measurements, supporting the validity of the visual assessment method.

Analysis of variance (ANOVA) was used to compare the results of x‐ray measurements and visual estimates. ANOVA is a statistical method that determines whether the differences observed between multiple groups or measurement techniques are statistically significant. To further assess where significant differences lie, Tukey's honestly significant difference (HSD) test was applied. This test enables the identification of specific pairs of means that differ significantly from one another, providing a more detailed understanding of the sources of variation between the measurement methods.

## RESULTS

Data from 55 patients were analysed, including 28 men and 27 women, with a mean age of 65.3 ± 10.3 years (range, 42.5–85.6 years). The mean preoperative PTS measured on radiographs was 7.9°, and the mean postoperative value was 5.5°, corresponding to an average change of 2.4° ± 2.5° (range, –5° to 7.6°).

**Figure 2:**
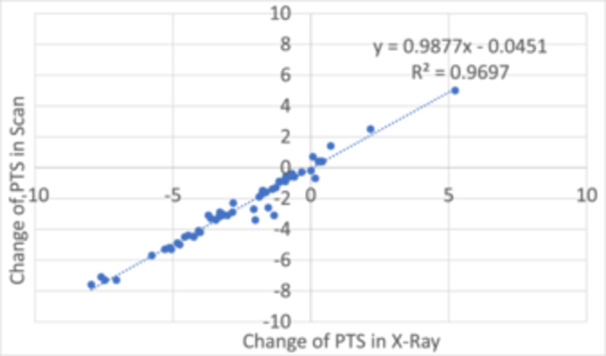
Change of posterior tibial slope (PTS) in scan versus x‐ray.

The reflected light (optical) scan measurements demonstrated close agreement with the radiographic measurements, with a median deviation of 0.3° and a maximum deviation of 1.8° (Figure 2). The deviations between surgeons' visual estimates and radiographic measurements were small, with mean differences of approximately 0.8° for Examiners 1 and 2, and 1.2° for Examiner 3. Similar levels of accuracy were observed when comparing surgeon estimates with optical scan measurements (Table 1).

**Table 1:** Deviation of inspector estimation to x‐ray and scan.

	Mean	Std. dev.	Min	Max	*p* value
Deviation of Inspector 1 versus x‐ray	0.8	0.5	0.01	2.0	0.06172
Deviation of Inspector 2 versus x‐ray	0.8	0.7	0.01	2.5	0.000016
Deviation of Inspector 3 versus x‐ray	1.2	0.7	0.01	3.0	0.0015
Deviation of scan versus x‐ray	0.3	0.3	0.01	1.8	0.40
Deviation of Inspector 1 versus scan	0.8	0.5	0.1	2	0.061
Deviation of Inspector 2 versus scan	0.9	0.7	0.1	2.3	0.000029
Deviation of Inspector 3 versus scan	1.1	0.7	0	2.7	0.00089

Abbreviation: Std. dev., standard deviation.

The ICC between optical scan and radiographic measurements was 0.99, indicating near‐perfect agreement. ICCs for visual estimates compared with radiographic measurements were 0.93 for Examiner 1, 0.96 for Examiner 2 and 0.88 for Examiner 3. All values exceeded the threshold for excellent reliability, confirming a high degree of consistency between visual and objective assessments.

**Figure 3:**
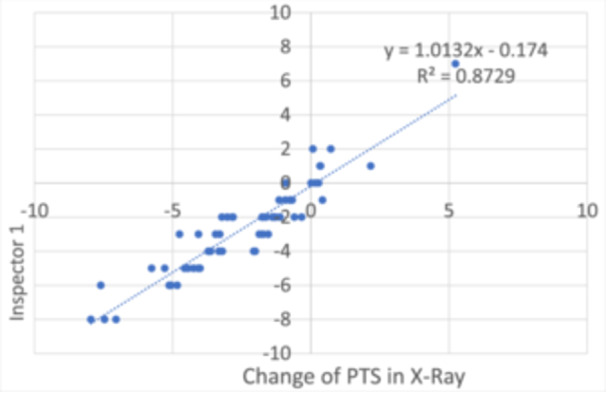
Change of posterior tibial slope (PTS) estimated by Surgeon 1 versus x‐ray.

**Figure 4:**
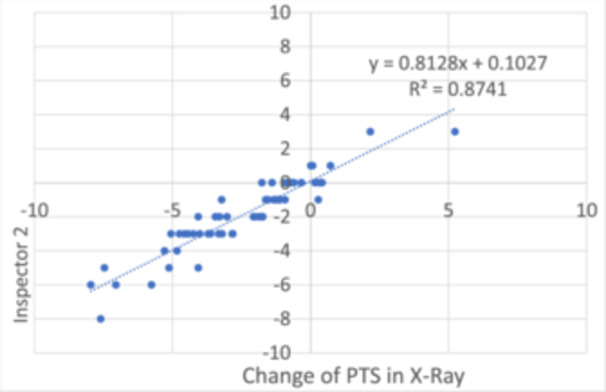
Change of posterior tibial slope (PTS) estimated by Surgeon 2 versus x‐ray.

**Figure 5:**
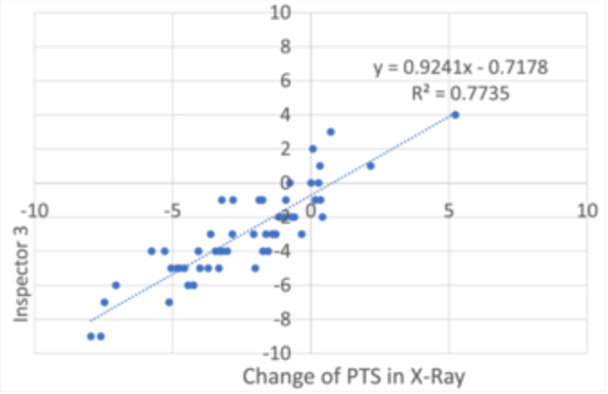
Change of posterior tibial slope (PTS) estimated by Surgeon 3 versus x‐ray.

Pearson correlation coefficients also demonstrated strong positive relationships between visual and radiographic measurements, ranging from 0.77 to 0.87 (Figures 3, 4, 5). Examiner 2 showed the strongest correlation, whereas Examiner 3 displayed the greatest variability, though all correlations remained within the range of strong agreement.

ANOVA revealed no statistically significant differences between radiographic and visual estimates, indicating comparable mean values across measurement methods. Tukey's HSD test confirmed the absence of significant pairwise differences among examiners and reference measurements (Tables 2 and 3).

**Table 2:** Analysis of variance (result details).

	SS	df	MS	
Between‐treatments	34.5	3	11.5	*F* = 1.8
Within‐treatments	1386.5	216	6.4	
Total	1421	219		

Abbreviations: df, degrees of freedom; MS, Mean Square; SS, Sum of Squares.

**Table 3:** Tukey's honestly significant difference (HSD) test.

	HSD_.05_ = 1.3 HSD_.01_ = 1.5	*Q*_.05_ = 3.7 *Q*_.01_ = 4.5
Inspector 1 versus x‐ray	0.15	*Q* = 0.43 (*p* = 0.99049)
Inspector 2 versus x‐ray	0.62	*Q* = 1.81 (*p* = 0.57694)
Inspector 3 versus x‐ray	0.47	*Q* = 1.38 (*p* = 0.76191)

## DISCUSSION

The main result of this study is that visual inspection of the tibial resection surface by experienced surgeons is sufficiently accurate to detect whether the PTS is reconstructed during UKA. Numerous studies have confirmed the importance of accurate slope reconstruction for postoperative function, stability and implant longevity [1, 8, 11, 13].

With an ageing population, the incidence of knee osteoarthritis continues to rise, leading to an increasing demand for knee replacement surgery [19]. The proportion of unicondylar prostheses has steadily grown, and in the past decade, approximately 30% of all knee replacements have been unicondylar [4]. Compared with TKA, UKA offers lower perioperative morbidity, shorter hospitalisation, faster recovery and greater knee mobility [15, 24]. Furthermore, UKA has been shown to be more cost‐effective than TKA [5].

Unlike TKA, which establishes new joint geometry, UKA aims to restore the native, pre‐arthritic anatomy. Therefore, precise reconstruction of the PTS is essential in UKA. Previous studies have demonstrated that the PTS affects sagittal stability, kinematics, flexion and implant wear [6, 8, 9, 11, 22, 25]. Deviations beyond a few degrees from the native slope may alter ligament tension, reduce ROM and accelerate polyethylene wear. A PTS between 4° and 8° is generally associated with favourable biomechanics and reduced wear, whereas extreme deviations, particularly beyond 9°, have been linked to implant failure [11, 12, 13, 25, 26].

Given its critical role, accurate intraoperative control of PTS is necessary. While robotic and navigated systems improve precision [7, 16], their use remains limited by cost and set‐up time [17]. Even with advanced instrumentation, intraoperative slope control remains challenging, as conventional tools do not allow precise measurement. Several studies have compared manual, navigated and robotic UKA and reported superior accuracy for robotic assistance but at the expense of efficiency and accessibility [7, 16, 17, 18].

In the present study, the accuracy of visual PTS estimation was evaluated against radiographic and optical references. The results showed excellent agreement between visual, radiographic and scan‐derived measurements, with ICCs ranging from 0.88 to 0.96. These findings indicate that experienced surgeons can reliably identify slope deviations within clinically acceptable limits. Visual inspection may therefore serve as a practical intraoperative alternative in settings where advanced technology is unavailable.

Several limitations of this study should be considered. First, TKA resections modified to simulate UKA resections were used, which may not fully reflect the intraoperative conditions of true UKA procedures. Although this approach enabled a broader range of slope variations for analysis, it may have introduced differences in bone morphology. Second, the sample size of 55 resections, while statistically adequate, limits subgroup analyses and generalisability. Third, all assessments were conducted in a laboratory environment, under optimal visibility and without the time constraints or distractions typical of surgery. As a result, the observed accuracy may represent a best case scenario. Fourth, although standardised imaging was used, radiographic measurements can still be affected by minor variations in projection or alignment. Lastly, the participating surgeons were highly experienced and familiar with the visual inspection technique, which may have positively influenced accuracy compared with less experienced operators.

Future studies should evaluate the reproducibility of these findings in real intraoperative settings and across different levels of surgical experience. It would be valuable to determine the learning curve for reliable visual PTS assessment and to compare intraoperative visual estimations directly with postoperative imaging. Investigating adjunct tools such as simple mechanical or digital aids that enhance visual estimation accuracy could further improve intraoperative slope control without the need for complex navigation systems.

## CONCLUSION

The intraoperative visual inspection of the tibial resection by experienced surgeons is suitable for assessing the reconstruction of the tibial slope during UKA.

## AUTHOR CONTRIBUTIONS

All authors contributed to the study conception and design. Material preparation, data collection and analysis were performed by Harun Hawi and Humam Hawi. The first draft of the manuscript was written by Harun Hawi, and all authors commented on previous versions of the manuscript. All authors read and approved the final manuscript.

## CONFLICT OF INTEREST STATEMENT

The authors declare no conflicts of interest.

## ETHICS STATEMENT

This retrospective study was approved by the local Ethics Committee in the University of Jena (Reg.‐Nr.: 2020‐2019_2‐Material).

## PATIENT CONSENT STATEMENT

Informed consent was obtained from all individual participants included in the study.

## PERMISSION TO REPRODUCE MATERIAL FROM OTHER SOURCES

No Material was used from other sources, thus no Permission was needed.

## Data Availability

The data that support the findings of this study are available on request from the corresponding author. The data are not publicly available due to privacy or ethical restrictions.
